# Antibiotic resistance profile of Gram-negative bacteria isolated from Lafenwa abattoir effluent and its receiving water (Ogun River) in Abeokuta, Ogun state, Nigeria

**DOI:** 10.4102/ojvr.v87i1.1854

**Published:** 2020-09-15

**Authors:** Samuel N. Akpan, Olubusola A. Odeniyi, Oluwawemimo O. Adebowale, Selim A. Alarape, Olanike K. Adeyemo

**Affiliations:** 1 Department of Veterinary Public Health and Preventive Medicine, Faculty of Veterinary Medicine, University of Ibadan, Ibadan, Nigeria; 2 Department of Microbiology, Faculty of Sciences, University of Ibadan, Ibadan, Nigeria; 3 Department of Veterinary Public Health and Preventative Medicine, College of Veterinary Medicine, Federal University of Agriculture, Abeokuta, Nigeria

**Keywords:** antibiotic resistance, multiple antibiotic resistance index, Gram-negative bacteria, abattoir effluent, surface water

## Abstract

Untreated abattoir effluent constitutes potential reservoir for transmission of pathogenic strains of multiple antibiotic-resistant bacteria by pollution of surface and ground water sources. This study was carried out to determine the antibiotic resistance and extended spectrum β-lactamase (ESBL) production profiles of Gram-negative bacteria isolated from effluent collected from Lafenwa municipal abattoir and its receiving surface water, Ogun River, in Abeokuta, Ogun state, Nigeria. Twelve effluent and 18 water samples were collected for this study. Total heterotrophic and coliform counts were estimated, bacterial identification was performed using standard culture-based procedures, whilst antibiotic resistance profiles of isolated bacteria against five antibiotics (ceftazidime, cefpodoxime, cefotaxime, ertapenem and amoxicillin-clavulanate) and detection of ESBLs were done using disk diffusion and double-disc synergy tests. A total of 54 Gram-negative bacteria were isolated, including *Salmonella* spp. (9), *Escherichia coli* (15), *Klebsiella* spp. (7), *Shigella* spp. (5), *Pseudomonas* spp. (12) and *Enterobacter* spp. (6). Both *Enterobacteriaceae* and *Pseudomonas* isolates (31% and 66.6%, respectively) were resistant to all selected antibiotics except ertapenem (98% susceptibility). Overall, 77% isolates had multiple antibiotic resistance index (MARI) values, but none of the antibiotic-resistant isolates showed evidence of ESBL production. The presence of multiple antibiotic-resistant isolates in the effluent and receiving water of Lafenwa abattoir suggests a major risk to public health and food safety. Current methods of waste disposal at the abattoir are unacceptable and greatly reduce the qualities of the processed meat and contaminate the environment. There is a need for improved abattoir waste management and water treatment strategies.

## Introduction

The water ecosystem is often degraded when the inherent potential of a water body to purify itself is lowered; hence, a river or stream is said to be polluted (Denise & John 2014). Pollution is the presence of significant quantity of contaminants in the environment. Waste management, hygiene and sanitation in Nigeria pose a big challenge, especially with many complex issues intertwined with much larger sociocultural problems (Adedeji & Adetunji 2011). The adequate facilities to ensure safe disposal of abattoir wastes in a manner that will not constitute a potential hazard to public, animal and environmental health are considered very essential and still underdeveloped in many developing countries (Nafarnda et al. 2012). Moreover, abattoirs in Nigeria have no facilities for waste treatment; wastes are either disposed on open dumps or are discharged into nearby streams, hence constituting an environmental menace (Adeyemo 2002). In particular, the Lafenwa Municipal abattoir effluents and wastes are channelled into Ogun River, which is used by indigenes of surrounding communities for drinking and domestic purposes, without prior treatment to remove bacteria and other noxious substances (Adebowale et al. 2016).

Abattoir effluent contains several million colony forming units (cfu) of total aerobic bacteria count and faecal coliforms (Omole & Longe 2008). Several previous studies have reported the isolation of pathogenic bacterial and fungal species, such as *Staphylococcus aureus, Streptococcus, Salmonella* spp., *Escherichia coli, Aspergillus, Mucor, Saccarhomyces* spp. and *Penicillium* spp., from abattoir wastewater (Adesomoye, Opera & Makinde 2006; Adebowale et al. 2016; Coker, Olugasa & Adeyemi 2001). Isolated pathogens threaten public and environmental health by introducing resistant strains or variants, which could migrate into ground or surface water, animals and other vectors. Multiple antibiotic-resistant *Enterobacteriaceae* from waste water and sludge of slaughterhouses have also been documented and intestinal contents/faeces of slaughtered animals at abattoirs, such as sheep, goats and cattle, are significant reservoirs (Martins Da Costa, Vaz-Pires & Bernardo 2006; Um et al. 2016). Multidrug resistance especially against antibiotics is becoming an emerging challenge to public, animal and environmental health, calling for its strict judicious and stewardship use in food animals.

Although few studies have reported the bacteriological assessment of Lafenwa abattoir effluent and its receiving water, none have focused on the antibiotic-resistant potential of these pathogens. Hence, this current study aimed at investigating this gap.

## Materials and methods

### Study area

The study area is located in Abeokuta North Local Government Area, Ogun state, Nigeria, with a total land mass of 808 km^2^ and a population of 201 329 (Nigeria Population Commission [NPC] 2006). The Lafenwa Municipal abattoir is situated on geographical coordinates 7° 10′ 0″ N and 3° 3′ 0″ E. The abattoir is a major slaughtering facility in Ogun state where a total number of 150–200 cattle are slaughtered daily for public consumption, and waste effluents are discharged directly into an adjoining water body (Ogun River). The slaughtering activities commence from 06:00 to 12:00 every day, except on Sundays (Adebowale et al. 2016).

### Sample collection

Effluent and water samples were collected between May and June 2019. At the abattoir, sampling points were divided into Point A (channel draining the trippery and guttery section) and Point B (main outflow channel discharging into the river) (Adebowale et al. 2016). On the river, the sampling points were divided into upstream (75 m before the point of inflow of abattoir effluent into the river), midstream (at the point of abattoir effluent release into river) and downstream (75 m after the point of effluent inflow) (Adebowale et al. 2016). In total, 12 effluent and 18 water samples were collected fortnightly over a 6-week period. Collected samples were transported on ice to the laboratory for microbial analysis.

### Bacterial isolation

Bacteria were isolated according to the process defined by Harrigan and McCance (1976). Firstly, 9 mL of sterile distilled water was dispensed into seven test tubes. Then, 1 mL of the effluent was dispensed into the first test tube and serially diluted at a ratio of 1:10. Water samples from the receiving surface water body were diluted serially at a ratio of 1:7. About 1 mL of each of the effluent and water samples was inoculated on aseptically prepared MacConkey agar (MA) (HiMedia Laboratories Pvt. Ltd, Mumbai, India), Eosine Methylene Blue (HiMedia India) and *Salmonella Shigella* agar (HiMedia India) using the pour plate technique. Plates were incubated at 35 °C ± 2 °C for 18 –24 hours.

The total heterotrophic and coliform counts were determined on nutrient agar (NA) and MA plates. The maximum limit of heterotrophic count for drinking water is 1.0 × 10² cfu/mL (Environmental Protection Agency [EPA] 2002), whereas the maximum limit for wastewater is 400 cfu/mL. Morphologically distinct colonies were sub-cultured twice on corresponding media. Pure colonies obtained were stored both on NA slants and nutrient broth containing 15% glycerol (volume/volume [v/v]), for further investigation.

## Phenotypic characterisation of bacteria

The pure isolates were characterised based on Gram status using the potassium hydroxide water (KOH) test. Using a sterile loop, a colony of overnight bacterial culture was gently mixed with a drop of 3% KOH (v/v) on a clean microscope slide. The loop was gently raised from the KOH-bacterium mixture to a height of 1 cm. A viscous drawing reaction indicated positivity of the test (Gram-negative reaction), and a non-viscous reaction showed that the isolate was Gram-positive (Powers 1995). Furthermore, biochemical characterisation of bacteria isolates was conducted using the catalase, urease, methyl red, Voges Proskauer, citrate and sugar fermentation tests.

## Antibiotic susceptibility test

The disc diffusion technique according to Bauer et al. (1996) was conducted to determine the antibiotic susceptibility pattern of isolates. Firstly, one colony of overnight bacterial culture was inoculated into normal saline and standardised to 0.5 McFarland. Then, previously prepared sterile Mueller Hinton (MH) agar plates (BioLab, Wadeville, Germiston, South Africa) were inoculated, carefully spread over the entire surface of the agar using sterile swab and allowed for 5 min to dry. Using a sterile forceps, following antibiotic discs – cefotaxime, cefpodoxime, amoxicillin-clavulanate, ceftazidime and ertapenem were placed onto the surface of the MH agar, and incubated at 35 °C ± 2 °C for 18–24 h. The zone of inhibition was read post-incubation and recorded following the Clinical and Laboratory Standards Institute guidelines (Clinical and Laboratory Standards Institute [CLI] 2014). Isolates showing resistance to any of the third-generation cephalosporins were further investigated for extended spectrum β-lactamase (ESBL) production.

## Detection of extended spectrum β-lactamase production

The detection of ESBLs producing bacteria was carried out using the Double-Disc Synergy Test Kit, following the Clinical and Laboratory Standards Institute (CLSI) (2014) guidelines. Bacteria isolates resistant to any of the third-generation cephalosporin were standardised to 0.5 McFarland. Subsequently, the standardised broth was gently spread over MH agar plates, and three antibiotics (ceftazidime, cefpodoxime and cefotaxime) were placed at a distance of 20 mm from amoxicillin-clavulanate (Augmentin) disc, which was placed at the centre. The plates were incubated at 35 °C ± 2 °C for 18–24 h. At the end of the incubation period, the plates were observed for isolates showing enhancement towards the Augmentin disc.

### Calculation of multiple antibiotic resistance indices

The multiple antibiotic resistance indices (MARI) were calculated according to the formula described by Krumperman (1983):

MARI=ab [Eqn 1]

where a = Total number of antibiotics to which an isolate shows resistance

b = Total number of antibiotics to which the isolate was exposed.

### Ethical considerations

This article followed all ethical standards for a research without direct contact with human or animal subjects.

## Results

The mean total heterotrophic and coliform counts of both the effluent from the two points and water samples from Ogun River were estimated and are presented in Table 1. The mean total heterotrophic counts (THC) for the effluent from sampling point B were higher (30.0 × 10^6^ cfu/mL) than that of sampling point A (13.5 × 10^6^ cfu/mL), with both points exceeding the maximum limit of 400 cfu/mL for wastewater. Also, THC for all the water samples collected from Ogun River exceeded the maximum limit of 1.0 × 10² cfu/mL for drinking water.

**TABLE 1:** Mean total heterotrophic and coliform counts of the abattoir effluents and its receiving water body (Ogun River).

Source	Total heterotrophic count (1 × 10^6^ cfu/mL)	Total coliform counts (1 × 10^6^ cfu/mL)
Effluent from sampling point A	13.5	15.0
Effluent from sampling point B	30.0	1.0
Upstream	0.5	0.11
Midstream	13.6	24.0
Downstream	5.2	15.0

**FIGURE 1:**
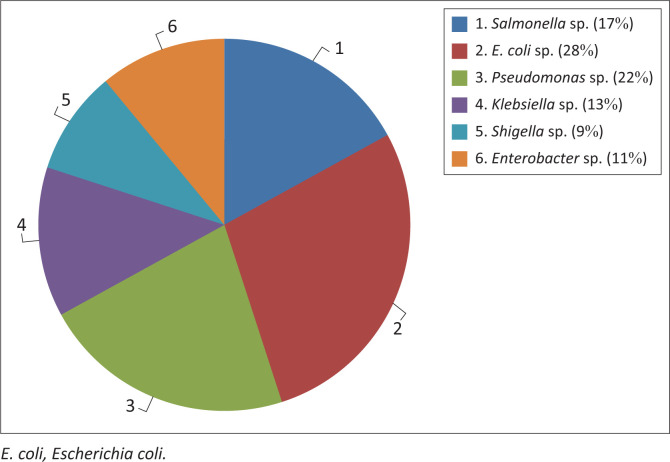
Frequency of occurrence of isolated bacterial species in the abattoir wastewater.

Pathogenic bacteria including *Salmonella, Pseudomonas, Enterobacter, Klebsiella* and *Shigella* were isolated from water sources used for meat processing and effluents of the abattoir. The frequency of occurrence of different types of bacterial species isolated from the abattoir wastewater is presented in Figure 1.

**FIGURE 2:**
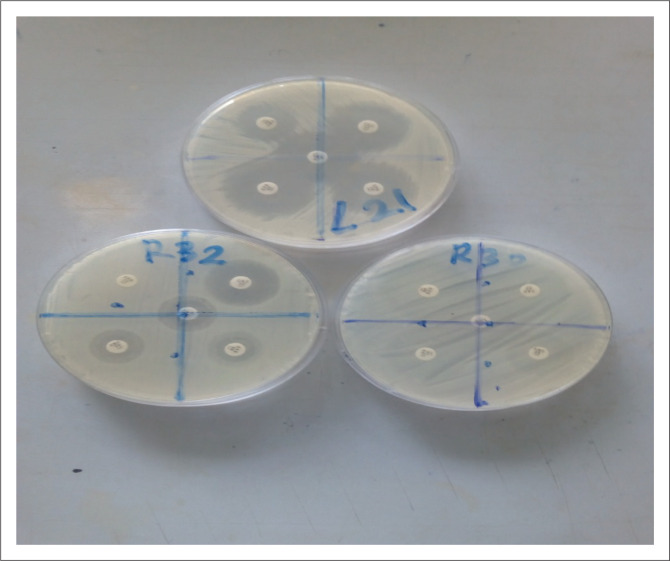
Cultured agar plates of isolates with zones of bacterial inhibition (L21, R32) and *Escherichia coli* resistance to antibiotic discs (R30).

**FIGURE 3:**
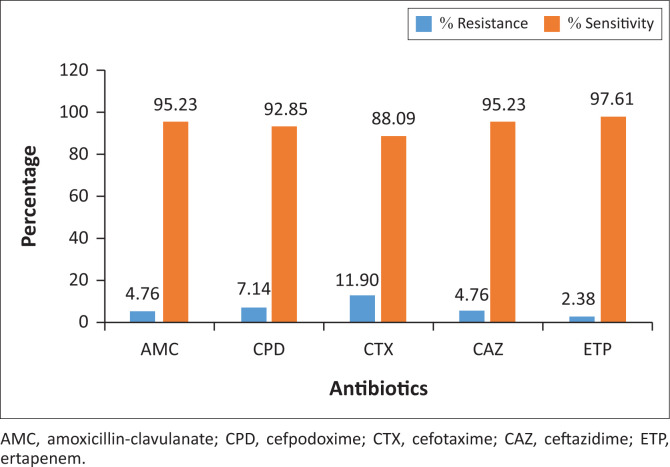
Antibiotic sensitivity and resistance pattern for *Enterobacteriaceae* isolates.

**FIGURE 4:**
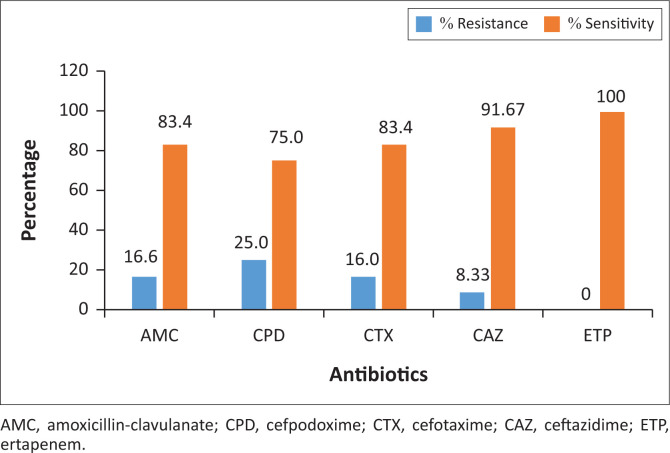
Antibiotic sensitivity and resistance pattern for *Pseudomonas* isolates.

The antibiotic sensitivity and resistance patterns of isolated Gram-negative bacteria were determined and are presented in Figures 2–4. The *Enterobacteriaceae* isolates showed resistance to amoxicillin-clavulanate (4.76%), cefpodoxime (7.14%), cefotaxime (11.90%), ceftazidime (4.76%) and ertapenem (2.38%). A total of 16.6% of *Pseudomonas* isolates were resistant to amoxicillin-clavulanate, 25% to cefpodoxime, 16.6% to cefotaxime and 8.33% to ceftazidime. Meanwhile, *Pseudomonas* isolates were 100% sensitive to ertapenem, suggesting a high potency of action of that class of antibiotics (carbapenems) against *Pseudomonas* organisms.

The MARI of isolates against the selected antibiotics are presented in Table 2. The study results showed that 77% MARI of the bacterial isolates were between values 0.2 and 1 (Table 2).

**TABLE 2:** Multiple antibiotic resistance indices of isolates to selected antibiotics.

Bacteria spp.	Source	Multiple antibiotic resistance indices (MARI)
*Salmonella*	MS	0.2
*E. coli*	MS	1.0
*E. coli*	E1	0.4
*Klebsiella*	MS	0.2
*Klebsiella*	MS	0.4
*Pseudomonas*	E2	0.4
*Pseudomonas*	E2	0.6
*Pseudomonas*	E1	0.6
*Pseudomonas*	E1	0.2
*Pseudomonas*	E1	0.4

*E. coli, Escherichia coli*; spp., species; MS, midstream; E1, effluent from sampling point A; E2, effluent from sampling point B; MARI, multiple antibiotic resistance indices.

## Discussion

This study revealed high heterotrophic and coliform counts in both the effluents from Lafenwa abattoirs and the receiving water body (Ogun River). The results of this study are similar to those of Umunnakwe, Akagba and Aharanwa (2013) who assessed the bacterial contents of the Aba River, where bacterial counts exceeded the permissible standards. A study by Adebowale et al. (2016), which investigated potential bacterial zoonotic pathogens at Lafenwa abattoir, also showed that mean total bacteria and coliform counts for the waste water and the receiving surface water exceeded the EPA (2002) and World Health Organization (WHO) (2004) maximum recommended and permissible limits of 400 cfu/mL and 200 cfu/mL, respectively. The high heterotrophic counts in the effluent and water samples indicated the presence of high levels of dissolved salts and organic micronutrients, which support the growth of microbial populations. The primary sources of these microorganisms are the wastes from the slaughtered animals. Also, the high counts of THC and total coliform counts (TCC) recorded in the midstream (13.6 × 10^6^ cfu/mL and 24 × 10^6^ cfu/mL) and downstream (5.2 × 10^6^ cfu/mL and 15 × 10^6^ cfu/mL) indicated a correlation between the bacteriological quality of the discharged effluents and the river water.

Total coliform counts for the water samples exceeded the maximum limits of 1.0 × 10² cfu/mL recommended by EPA (2002) in drinking water. This is indicative of faecal contamination at different points of river sampling. All water samples were found to have coliform counts, which exceeded surface water limits of 200/100 mL, and wastewater limits of 400/100 mL, indicative of diffuse faecal pollution of Ogun River. This is in agreement with the assertion of Adeyemi and Adeyemo (2007) that the presence of faecal coliforms attributed to poor sanitary hygiene standards and lack of efficient waste management control systems in rivers and streams is very common in developing countries.

The results of isolated bacteria showed that *E. coli* had the highest percentage frequency of 28%. This result tallies with the findings of Nwankwo, Magaji and Tijani (2015) with a reported frequency of 30.5%, which was also the highest amongst all the bacterial isolates. However, the total heterotrophic and coliform water samples obtained downstream showed lesser values, suggestive of dilution effect with respect to time, surface area and rainfall (Adebowale et al. 2016). The occurrence of other pathogenic bacteria in abattoir water sources used for meat processing and effluents, such as *Salmonella, Pseudomonas, Enterobacter, Klebsiella* and *Shigella*, which were also isolated in this study, has also been widely reported (Galvin et al. 2010; Nafarnda et al. 2012). These bacterial organisms have been implicated as the causes of Typhoid fever, shigellosis, pneumonia, meningitis, urinary tract infections (UTI), cellulitis and neonatal sepsis (Neu 1985).

As shown in Figure 4, isolated *E. coli* from the receiving water showed resistance to all the five selected antibiotics, an indication of strong inactivation or hydrolysing action against the different classes of antibiotics (β-lactam/β-lactamase inhibitors, carbapenems and cephems). Antibiotic resistance in *E. coli* is of particular concern because it is the most common Gram-negative pathogen in humans, multidrug-resistant strains and is easily transferable to other strains (Rasheed et al. 2014). When an organism becomes resistant to first-, second- and third-line antibiotic agents, it becomes a nightmare for the physician and the duration of an infection becomes prolonged (Kim & Aga 2007; Schmidt 2002).

The MARI showed that 77% of the bacteria isolated in this study were between values 0.2 and 1 (Table 2). According to Chitanand et al. (2010), MARI values less than 0.2 are considered to be an original strain resistance to an antibiotic on first exposure whilst values above 0.2 infer that the effluent and receiving water are high-risk point sources (points where the risk of entry, contact or infection with pollutant is the highest, which are basically the points at the river site where pollution is high) (Mthembu 2008). Therefore, the MARI values ≥ 0.2 obtained infer that the slaughtered animals from which the wastes were obtained have been exposed to frequent antibiotic therapy over time, leading to bacterial resistance to selected antibiotics. This is a pointer to the indiscriminate use of antibiotics on livestock by farmers for growth promotion, prophylactics and therapeutic purposes. Here, the major incriminated stakeholders are uneducated nomadic cattle rearers who serve dual functions of producers and health personnel to large livestock herds, some in the hinterlands, with no form of training or monitoring on the use of antibiotics. These animals are later sold to buyers and slaughtered in abattoirs, with attendant risks involved (Alhaji 1976). None of the isolates tested in this study were positive to the DDST for ESBL production, suggesting that other mechanisms of bacterial resistance could be responsible. Furthermore, none of the Gram-negative bacteria isolates (*Salmonella* spp., *E. coli, Klebsiella* spp., *Shigella* spp., *Pseudomonas* spp. and *Enterobacter* spp.) from downstream showed resistance to the selected antibiotics, which may have been because of the possibility of river purification by dilution (as a result of distance from the point source) and rainfall.

## Conclusion

Multidrug-resistant bacteria are difficult to treat, thereby increasing the spread of resistance and complicating efforts to combat them in animals and humans. This study exposed the increasing trend and prevalence of multidrug-resistant bacteria and a high risk of transfer of multiple resistant genes to other microbial populations in the aquatic ecosystem. Thus, Ogun River is a reservoir and foci for the spread of multidrug-resistant bacteria to aquatic life, animals and humans.

The multiple antibiotic-resistant pathogens isolated from high MARI reported in this study should alert government, public health and veterinary authorities about the dangers of irresponsible use of antibiotics in animals and the rationalisation of antibiotics use in Nigeria. Also, the role of animal wastes in the spread and maintenance of multiple antibiotic-resistant strains of pathogenic Gram-negative bacteria through the water environment as shown in this study calls for the attention of relevant authorities for appropriate action in water sanitation and management, proper effluent treatment and disposal, policy formulation and enforcement, protection of public and ecosystem health, and curbing the menace of antimicrobial resistance in Nigeria.
